# International cost-effectiveness analysis in osimertinib after chemoradiotherapy in stage III EGFR-mutated non-small cell lung cancer

**DOI:** 10.3389/fpubh.2025.1698562

**Published:** 2025-10-29

**Authors:** Diya Tang, Xi Zou, Chaochao Wei, Xiaoyu Zhang

**Affiliations:** ^1^Department of Oncology, The First Affiliated Hospital, Hengyang Medical School, University of South China, Hengyang, China; ^2^Department of Oncology, Xiangya Hospital, Central South University, Changsha, China; ^3^Department of Pulmonary and Critical Care Medicine, Affiliated Hainan Hospital of Hainan Medical University, Haikou, China; ^4^Key Laboratory of Emergency and Trauma of Ministry of Education, Hainan Medical University, Haikou, China; ^5^NHC Key Laboratory of Tropical Disease Control, Hainan Medical University, Haikou, China

**Keywords:** EGFR-mutated, non-small cell lung cancer, osimertinib, chemoradiotherapy, cost-effectiveness

## Abstract

**Background:**

The international Phase 3 LAURA trial (NCT03521154) demonstrated that the use of osimertinib following chemoradiotherapy markedly improved survival outcomes in unresectable stage III NSCLC with epidermal growth factor receptor (EGFR) mutations. Considering the high cost of targeted therapy, the popularization of osimertinib in clinical practice should be considered comprehensively in terms of cost and efficacy. This study was to investigate the cost-effectiveness of osimertinib for unresectable stage III EGFR-mutated NSCLC without disease progression after chemoradiotherapy from the perspective of payers in the USA and China.

**Methods:**

The main health outcomes were evaluated by measuring life-years (LYs), quality-adjusted life-years (QALYs), incremental cost-effectiveness ratio (ICER), and incremental net health benefit (INHB). An integrated Markov model with three separate health states over a 15-year horizon was established. The sensitivity of the model was assessed, and subgroup analyses were conducted.

**Results:**

Compared with placebo in stage III EGFR-mutated NSCLC after chemoradiation, osimertinib [$898,107 (3.70 QALYs) and $49,565 (3.49 QALYs)] increased costs (efficacy) by $178,953 (0.56 QALYs) in the USA and $17,872 (0.51 QALYs) in China. The corresponding ICERs were $322,308/QALY and $35,186/QALY, respectively, with an INHB of −0.63 and 0.06 QALYs. The sensitivity analysis showed that the results were influenced significantly by progression-free survival.

**Conclusions:**

In China, treatment with osimertinib rather than placebo appears to be an effective and economically accessible option for patients with stage III EGFR-mutated NSCLC with no disease progression after chemoradiotherapy. This applied especially to the eastern and central economic regions of China but not the USA currently.

## 1 Background

Lung cancer remains the most common malignancy in the world with approximately 2.5 million new diagnoses and over 1.8 million deaths in 2022, with a 5-year survival of below 20% (1). Non-small cell lung cancer (NSCLC) accounts for about 85% of these diagnoses, with adenocarcinoma forming 40% of these cases and representing the most prevalent subtype since 2020 (2, 3). Stage III NSCLC shows a high degree of heterogeneity, and treatments are thus divided into three categories, namely, resectable, potentially resectable, and unresectable, depending on appropriateness of radical surgery (4, 5). Over 20% of patients have unresectable stage III locally advanced disease when diagnosed, for which the recommended treatment is concurrent chemoradiotherapy (CCRT) (6–9). The introduction of immunotherapy in 2017 has provided a new treatment model for these patients, where it is used as consolidation therapy in patients without progression following chemoradiotherapy, markedly improving patient survival (10–12). Approximately one-third of patients with unresectable stage III NSCLC treated with chemoradiotherapy have mutations in the epidermal growth factor receptor (EGFR) worldwide (13, 14). In China and other East Asian countries, EGFR mutations were detected in ~30% to 50% of patients with lung adenocarcinoma, whereas in the USA, the mutation rates were observed in only 8% to 21% (15).These patients have been found to have shorter or similar progression-free survival (PFS) compared with those without mutations as although there is a lower risk of treatment failure, the incidence of distant metastasis is higher, a finding subsequently confirmed in several real-world studies (16–18). Therefore, the control of systemic metastasis remains crucial for improving the long-term prognosis of these patients.

Osimertinib, a tyrosine kinase inhibitor (TKI) that inhibits EGFR, can effectively block the effects of various EGFR mutations, including deletions of exon 19 and the L858R substitution in exon 21 (19). It is approved for use as a postoperative adjuvant therapy for early-stage resectable lung cancers as well as a first-line therapy for advanced NSCLC with mutated EGFR or in combination with platinum-based chemotherapy (20). Due to its remarkable efficacy and minimal side effects in lung adenocarcinoma, osimertinib has become one of the most prescribed targeted drugs in recent years (21).

Addressing the huge unmet treatment need for patients with EGFR-mutated stage III unresectable NSCLC, the LAURA trial represents the first international Phase 3 clinical trial to directly compare the safety and efficacy of osimertinib with placebo in these patients after chemoradiotherapy, potentially establishing osimertinib as a new standard of care (20). The trial showed that osimertinib significantly improved PFS in cases without progression during or after chemoradiotherapy, with an estimated median PFS of 38.9 months vs. 7.3 months [hazard ratio (HR), 0.19; 95% confidence interval (CI), 0.12 to 0.29], resulting in an 81% reduction in disease progression and death (20). Although overall survival (OS) data have not yet reached maturity, interim analyses a trend towards OS benefit in the osimertinib group (HR, 0.81; 95% CI, 0.42 to 1.56), with a decrease of 19% in the risk of death. The PFS benefit of osimertinib over placebo was consistent across all key subgroups. Radiation pneumonitis was the most frequently reported treatment-related adverse event (TRAE) in both groups, the majority of which were low-grade, non-serious, and manageable, with no grade 4 or 5 events observed.

The LAURA trial has transformed the treatment landscape for these patients, suggesting the potential of osimertinib therapy.

Over the past few decades, despite a global decline in the overall incidence and mortality of lung cancer in most countries, the burden of lung cancer has been largely driven by changes in the morphological subtype patterns, with a continuous increase in the incidence of adenocarcinoma (22). This necessitates further accurate identification and in-depth analysis of differences among subtypes by clinical researchers, as well as increased attention from healthcare policymakers to the clinical accessibility of personalized treatment strategies. However, the high cost of targeted therapies and the large patient population render it unaffordable for both individuals and healthcare systems. It is thus necessary and urgent to conduct an economic analysis of the clinical benefits associated with specific targeted therapies. Here, the relative cost-effectiveness of osimertinib vs. placebo was assessed as consolidation treatment for unresectable stage III NSCLC post-chemoradiotherapy, from the perspectives of healthcare systems in high- and middle-income countries, represented by the USA and China, respectively.

## 2 Materials and methods

This study was entirely based on previous research and publicly available disease progression and therapy data, and it did not include any new research involving human participants or animals by any of the authors and therefore does not require approval from an independent ethics committee. The analysis followed the Consolidated Health Economic Evaluation Reporting Standards 2022 (CHEERS 2022) Statement, as shown in Supplementary Table S1.

### 2.1. Patient population and intervention

Using a simulated patient cohort with identical characteristics to that used in the LAURA clinical trial, patients with sensitizing EGFR mutations and stage III unresectable NSCLC without progressive disease post-chemoradiotherapy were hypothetically randomized in a 2:1 ratio to be treated with either osimertinib or placebo, with a daily dose of 80 mg until disease progression, death, or discontinuation for other reasons (20). Imaging assessments were conducted every 8 weeks. For consistency, any patient in the osimertinib or placebo group who showed progressive disease (PD) received subsequent systemic anticancer therapy [42 cases (29.4%) and 57 cases (78.1%)], respectively. The majority of patients in both groups received subsequent osimertinib treatment after disease progression, as reported in the corresponding trial (20). Patients who did not receive further treatment were considered to have undergone the best supportive care (BSC) before death, and none of those who died of treatment-related causes were included. Supplementary Table S2 details the drug dosages and unit prices.

### 2.2. Model design

An integrated Markov model was utilized to simulate the disease course of the patients in both cohorts using TreeAge Pro (version 2022). The model included three states of health, specifically, PFS, PD, and death. At the start of treatment, all patients fell into the PFS category, while after receiving osimertinib or placebo, they either remained in the PFS category or moved to the next categories, namely, PD or death (Supplementary Figure S1).

The Markov model had a cycle length of 4 weeks and a horizon of 15 years, during which it was anticipated that over 99% of patients would die within this time frame. Key outcomes predicted included life years (LYs), quality-adjusted life years (QALYs), the incremental cost-effectiveness ratio (ICER), and the incremental net health benefit (INHB) for the two interventions. Based on previous literature with an international perspective, the medical benefits and costs were discounted by 5% and 3%, respectively, each year (23, 24). The willingness-to-pay (WTP) thresholds were set at $150,000 for the USA and $39,632 [three times the per capita gross domestic product (GDP) in 2024] for China, with all costs expressed in US dollars (25, 26).

Patient survival curves were utilized to calculate the likelihood of change between the different states of health. Due to the lack of precise individual patient information in the study, survival information was extracted from the OS and PFS Kaplan-Meier (KM) curves reported by Hoyle et al., using the Get Data Graph Digitizer (version 2.26) (27). After the integration of this patient survival information, a parametric survival model was established (Supplementary Figure S1). According to the Akaike and Bayesian information criteria, visual analysis was used to select the Weibull distribution as the most suitable parameter distribution among the Exponential, Log-normal, Log-logistic, Weibull, and Gompertz distributions for reconstructing the model parameters (Supplementary Figure S2, Supplementary Table S3) (28). Subsequently, R Studio (version 1.2.5042) was used to obtain the specific distributions of the γ (scale) and λ (shape) parameters (Table 1).

**Table 1:** Input parameters.

**Variable**	**Baseline value (range)**	**Reference**	**Distribution**
Clinical parameters			
**Weibull survival model for osimertinib**			
OS	Scale = 0.000005603, Shape = 2.931	–	–
PFS	Scale = 0.033281, Shape = 0.849111	–	–
**Weibull survival model for placebo**			
OS	Scale = 0.0001721, Shape = 2.050	–	–
PFS	Scale = 0.12138, Shape = 0.96416	–	–
**Rate of post-discontinuation therapy**			
Osimertinib group	0.294 (0.235-0.353)	(19)	Beta
Placebo group	0.781 (0.625-0.937)	(19)	Beta
**Risk for main AEs in osimertinib group**			
Diarrhea	0.014 (0.0112–0.0168)	(19)	Beta
Radiation pneumonitis	0.014 (0.0112–0.0168)	(19)	Beta
Pneumonitis	0.014 (0.0112–0.0168)	(19)	Beta
**Risk for main AEs in placebo group**			
NA	NA	–	–
**Cost, $/per cycle (The United States)**			
**Cost of treatment**			
Osimertinib	18,034 (14,427.2–21,640.8)	(29)	Gamma
**Cost of AEs**			
Osimertinib group	351 (280.8–421.2)	(33)	Gamma
Placebo group	NA	-	-
Laboratory	609 (487.2–730.8)	(28)	Gamma
Radiological test	1,765 (1412–2118)	(28)	Gamma
Best supportive care	3,728 (2982.4–4473.6)	(28)	Gamma
Palliative care per patient	14,532 (11625.6–17438.4)	(28)	Gamma
Discount rate	0.03 (0–0.05)	(23, 24)	Uniform
**Cost, $/per cycle (China)**			
**Cost of treatment**			
Osimertinib	685 (548–822)	Local hospital	Gamma
**Cost of AEs**			
Osimertinib group	48 (38.4–57.6)	(30, 32)	Gamma
Placebo group	NA	–	–
Laboratory	609 (487.2–730.8)	(29)	Gamma
Radiological test	1,765 (1,412–2,118)	(29)	Gamma
Best supportive care	467 (373.6–560.4)	(30, 31)	Gamma
Palliative care per patient	2,349 (1,879.2–2,818.8)	(30, 31)	Gamma
Discount rate	0.05 (0–0.08)	(23, 24)	Uniform
**Utility and disutility**			
Utility of PFS	0.791 (0.6328–0.9492)	(29)	Beta
Utility of PD	0.653 (0.5224–0.7836)	(29)	Beta
Disutility of diarrhea	0.050 (0.040–0.060)	(37)	Beta
Disutility of radiation pneumonitis	0.090 (0.072–0.108)	(38)	Beta
Disutility of pneumonitis	0.090 (0.072–0.108)	(38)	Beta

NA, the sample size was unavailable or too small and was not further calculated. OS, overall survival; PFS, progression-free survival; PD, disease progressed; AEs, adverse events.

### 2.3. Costs and utility

The investigation included only direct medical costs, and indirect and hidden medical costs were excluded. The direct medical costs covered major interventional drugs, necessary laboratory and radiological tests, BSC, palliative care, and management of TRAEs (Table 1). The drug prices aligned with real hospital data and online queries of drug prices (29). Other direct medical costs were derived from the existing literatures (30–35). The analysis included TRAEs of grade 3 or above with a frequency of ≥1%. Notably, no grade 4/5 TRAEs were reported. All costs were corrected to the 2024 prices in terms of the US Consumer Price Index (CPI); in contrast, due to government regulation, drug prices in China did not require inflation adjustment to ensure cost stability (36). The costs were all calculated in US dollars based on the exchange rate of $1 = ¥7.2478 (June 2024).

Health utility values reflect patients' health-related quality of life (HRQOL) during the natural course of the disease, ranging from 0 (worst) to 1 (perfect) health status. As the LAURA trial did not provide similar information, health utility values of 0.791 and 0.653 were assigned for PFS and PD, respectively, based on previous studies with adjustment for the disutility values of adverse events (AEs) (30). These AE-associated disutility values were derived from previous reports (37, 38). It was found that the cost data conformed to a gamma distribution, while the utility values and AE incidence confirmed to a beta distribution (39).

### 2.4. Statistical analysis

Various methods for sensitivity assessments were used to elucidate the performance of the integrated model and the effects of variables on the outcomes. One-way sensitivity analysis covered extreme changes in each model parameter. The parameters were randomly altered by 20% within the range of their baseline values to identify parameters having a significant impact on the ICER value (40). A tornado diagram was used for displaying the importance of each parameter on the model outcomes. To simulate the impact of simultaneously changing several uncertain parameters that varied randomly within their distribution ranges on the ICER, probabilistic sensitivity analysis (PSA) was performing using 10,000 Monte Carlo simulations, and the results were displayed in scatter plots and cost-effectiveness acceptability curves to assess the cost-effectiveness of osimertinib relative to placebo (40). Additionally, the potential effects of different patient subgroup characteristics on the results were evaluated using targeted subgroup analyses of stratified patients, following the method of Hoyle et al. (41).

## 3 Results

### 3.1. Base-case analyses

The mature model with a 15-year projection showed that the life expectancy for patients with sensitising EGFR mutations stage III unresectable NSCLC in the USA and China, post-chemoradiotherapy and without disease progression increased by 0.45 LYs (5.40 months) and 0.39 LYs (4.68 months) respectively with osimertinib consolidation therapy relative to placebo. In terms of improvements in quality of life, in the USA and China, osimertinib incurred additional costs (efficacy) of $178,953 (0.56 QALYs) and $17,872 (0.51 QALYs) compared to placebo, respectively. Using a WTP threshold of $150,000/QALY (USA) and 39,632/QALY (China), the respective ICERs were 322,308/QALY and 35,186/QALY, with an INHB of−0.63 and 0.06 QALYs. The detailed results are presented in Table 2.

**Table 2:** Results of the base case analysis.

**Treatment**	**Total cost, $**	**Overall LYs**	**Overall QALYs**	**ICER, $**		**INHB, QALY**
				**Per LY**	**Per QALY**	
**The United States**						
Placebo	719,154	4.66	3.15	–	–	–
Osimertinib	898,107	5.11	3.71	401,133	322,308	−0.63
**China**						
Placebo	31,692	4.40	2.98	–	–	–
Osimertinib	49,565	4.80	3.49	45,335	35,186	0.06

LYs, life-years; QALYs, quality-adjusted life-years; ICER, incremental cost-effectiveness ratio; INHB, incremental net health benefits.

### 3.2. Sensitivity and subgroup analyses

**Figure 1:**
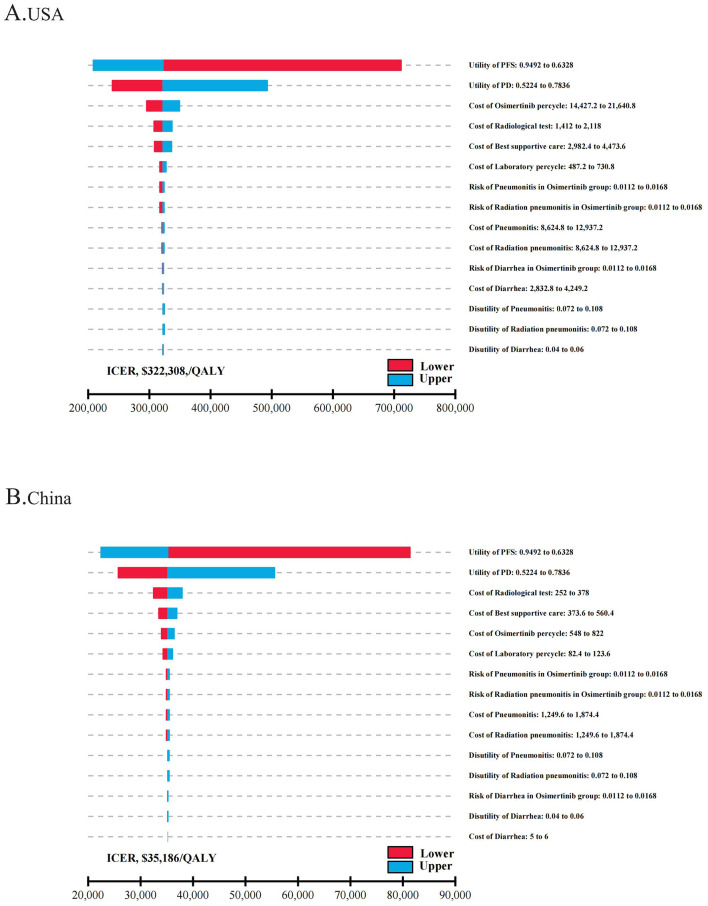
The one-way sensitivity analyses for osimertinib versus placebo strategy in the USA **(A)** and China **(B)**. PFS, progression-free survival; PD, progressive disease; ICER, incremental cost-effectiveness ratio; QALY, quality-adjusted life-year.

Tornado diagrams were utilized to display parameters that the one-way sensitivity analyses had shown to significantly influence the ICER values (Figure 1) The results indicated that in both the USA and China, the utility value of the patient's health status was the primary factor affecting the ICER, surpassing the cost of osimertinib and other follow-up expenses. The PFS utility value had the most significant impact on the ICER. In the USA, when the PFS utility value ranged from 0.6328 to 0.9492, the ICER per QALY was between $208,346 and $711,474. Regardless of parameter changes, the ICER values were consistently greater than the WTP threshold of $150,000/QALY. However, in China, when the PFS utility value rose to its upper limit, the ICER per QALY decreased to $22,465, indicating that osimertinib provided greater clinical benefits at a lower cost. When the PFS utility value dropped to its lower limit, the ICER increased to $81,368/QALY, exceeding the threshold of $39,632/QALY. Then we conducted a scenario analysis based on plausible ranges of health-utility values reported in NSCLC populations (42). Across utility scenarios A–C, all ICERs exceeded the WTP threshold of $150,000/QALY across all parameter settings In the United States. In China, only under the most conservative utility set (Scenario C) did the ICER ($43,942/QALY) exceed the prespecified threshold of $39,632/QALY (Supplementary Table S4). Other parameters, such as the cost of osimertinib, laboratory and radiological tests, and BSC costs, had a relatively limited impact on the model inputs.

**Figure 2:**
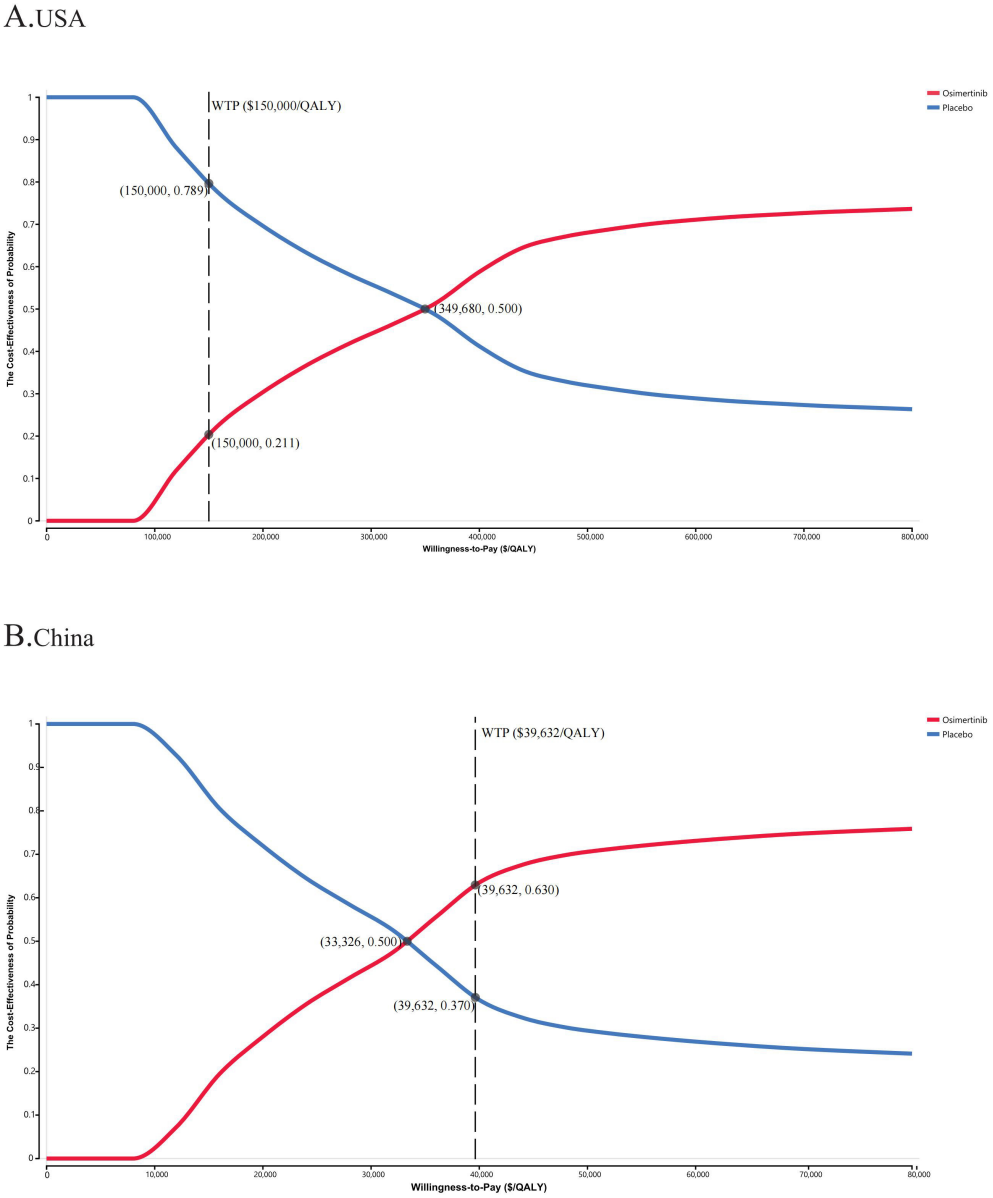
The cost-effectiveness acceptability curves for osimertinib versus placebo strategy in the USA **(A)** and China **(B)**. WTP, willingness-to-pay; QALY, quality-adjusted life-year.

The PSA results indicated that patients treated with osimertinib consolidation therapy had a 21.1% likelihood of it being a more cost-effective option, compared with 50.0% for the placebo, at the WTP thresholds of $150,000/QALY and $39,632/QALY, respectively (Figure 2 and Supplementary Figure S3). The cost-effectiveness acceptability curves indicated a strong correlation between economic advantage and increasing WTP thresholds. When the WTP thresholds increased to ~$400,000 in the USA and $45,000 in China, respectively, the probability of cost-effectiveness approached 60% in both settings, suggesting that the WTP threshold significantly influenced the chance of a specific treatment strategy becoming the primary option.

Subgroup analyses in the United States indicated that, aside from patients with the L858R EGFR mutation and those aged 65 years or older, sensitivity analyses revealed ICER values exceeding the WTP thresholds in nearly all subgroups, correlating with negative INHBs (Table 3). In the subgroup aged 65 years and older, osimertinib offered an additional 0.45 QALYs (0.21 LYs) compared to placebo, resulting in an ICER of $134,526 and an INHB of 0.05 QALYs. For the L858R EGFR mutation subgroup, the additional cost (efficacy) of osimertinib compared to placebo was $66,875 (0.46 QALYs), with corresponding ICERs of $146,821/QALY and an INHB of 0.01 QALYs. Conversely, in China, ICER values below WTP thresholds were observed across all patient subgroups, with the probability of osimertinib being cost-effective exceeding 50% in most analyzed subgroups. Osimertinib demonstrated superior cost-effectiveness and emerged as the optimal treatment for patients with unresectable stage III EGFR-mutated NSCLC without disease progression after chemoradiotherapy.

**Table 3:** Results of subgroup analyses.

**Subgroup**	**PFS HR (95% CI)**	**ICER, $/QALY**		**INHB, QALYs**		**Cost-effectiveness probability of osimertinib**	
		**The United States**	**China**	**The United States**	**China**	**WTP at $150,000/QALY**	**WTP at $39,632/QALY**
**Sex**							
Male	0.26 (0.15–0.46)	224,199	32,834	−0.25	0.08	30.9%	63.0%
Female	0.21 (0.13–0.34)	293,430	34,497	−0.51	0.06	23.7%	54.8%
**Age**							
< 65years	0.16 (0.10–0.26)	366,838	36,244	−0.85	0.05	17.4%	49.9%
≥65years	0.33 (0.19–0.57)	134,526	30,657	0.05	0.09	63.7%	69.1%
**Smoking history**							
Current or former	0.26 (0.14–0.48)	224,199	32,834	−0.25	0.08	31.2%	62.6%
Never	0.22 (0.14–0.34)	279,242	34,157	−0.45	0.06	24.4%	55.3%
**Stage**							
IIIA	0.28 (0.15–0.52)	197,715	32,194	−0.18	0.08	35.8%	65.9%
IIIB or IIIC	0.21 (0.13–0.33)	293,430	34,497	−0.51	0.06	24.2%	54.8%
**EGFR mutation**							
Exon 19 deletion	0.17 (0.10–0.29)	351,837	35,888	−0.77	0.05	19.2%	49.6%
L858R mutation	0.32 (0.19–0.56)	146,821	30,957	0.01	0.09	56.2%	68.4%
**Chemoradiotherapy**							
Concurrent	0.25 (0.17–0.36)	237,702	33,159	−0.30	0.08	29.6%	61.7%
Sequential	NA	NA	NA	NA	NA	NA	NA
**Response to previous CRT**							
Complete response	NA	NA	NA	NA	NA	NA	NA
Partial response	0.20 (0.11–0.34)	307,786	34,840	−0.57	0.06	21.9%	52.8%
Stable disease	0.18 (0.10–0.30)	336,993	35,535	−0.70	0.06	20.8%	50.8%
Not evaluable	NA	NA	NA	NA	NA	NA	NA

NA, The sample size was unavailable or too small and was not further calculated. CI, confidence interval; PFS HR, progression-free survival hazard ratio; ICER, incremental cost-effectiveness ratio; INHB, incremental net health benefits; QALY, quality-adjusted life-year; WTP, willingness-to-pay; CRT, chemoradiotherapy.

## 4 Discussion

EGFR mutations are active in all stages of NSCLC and represent the most common oncogenic mutations associated with stage III unresectable locally advanced NSCLC (43). To date, CCRT followed by immunotherapy consolidation remains the standard treatment. However, the effectiveness of immunotherapy in these patients remains unclear (44, 45). Despite better response rates to radical CCRT and lower local recurrence rates compared to patients with wild-type EGFR, they exhibit higher rates of distant metastasis, particularly to the central nervous system (16, 18). Over the past decade or so, the identification of specific driver mutation genes and the development and upgrading of targeted therapies has profoundly affected the treatment paradigm for NSCLC patients.

Osimertinib is one of the most effective new EGFR inhibitors and can inhibit both EGFR mutations and T790M resistance mutations, selectively targeting the EGFR tyrosine kinase and leading to significant and sustained tumor regression(19). According to the latest research data, osimertinib consolidation therapy can markedly extend PFS in these patients relative to placebo without significant toxicity, demonstrating better efficacy and safety, and greatly improving the quality of life of the patients. However, while providing clinical benefits and improving quality of life, these treatments inevitably impose a significant social burden and macroeconomic cost on healthcare systems worldwide. Additionally, in recent years, global guidelines have rarely addressed the issue of the costs associated with new anticancer drugs or treatments. Even for approved anti-cancer drugs, affordability is a key factor in determining their accessibility in clinical practice. Therefore, it is necessary to conduct economic evaluations of new treatments.

Given that the clinical evidence for the use of osimertinib consolidation therapy in these patients is relatively new, there is an absence of evidence on cost-effectiveness. The present study evaluated the cost-effectiveness of osimertinib consolidation therapy relative to placebo in these patients based on the most recent evidence and the healthcare systems of the USA and China. The economic analyses show that the efficacy of osimertinib treatment relative to placebo exceeded 0.5 QALYs. In the USA, the total treatment cost for the osimertinib group was $898,107, with an ICER of $322,308/QALY and a corresponding INHB of −0.63 QALYs. For Chinese patients, the total treatment cost for the osimertinib group was $49,565, with an ICER of $35,186/QALY and a corresponding INHB of 0.06 QALYs. From the perspective of healthcare services in both countries, the use of osimertinib relative to placebo for these patients is a cost-effective option in China but not in the USA, suggesting that patients in China will be more inclined to choose osimertinib as their first-line treatment. It also highlights the importance of local cost-effectiveness analysis that can be tailored to a country or specific region.

Although China and the United States bear the largest shares of global cancer-related economic costs, their per-capita health expenditure diverges substantially, with China at $672.5 and the United States at $12,434.4 in 2022 (46, 47). The US healthcare system is predominantly driven by private insurance, emphasizing market orientation and efficiency. Drug prices are largely determined by market competition, with pricing and reimbursement policies shaped by rigorous pharmacoeconomic evaluations. Reimbursement negotiations mainly focus on cost containment, often favoring established therapies with proven effectiveness and lower budget impact. Centers for medicare services may prompt pharmaceutical companies in the USA to re-evaluate their pricing strategies, with tiered pricing emerging as a potential approach to enhance both cost-effectiveness and market competitiveness. In contrast, China's healthcare system is primarily government-led, with drug prices set through state regulation and national policy. Within the constraints of the national health insurance budget, reimbursement negotiations aim to balance the sustainability of the fund with population access, thereby promoting more efficient resource allocation. Consequently, regulatory approval and inclusion in the national reimbursement drug list will be an essential step to enhance access to innovative therapies in China. Therefore, differences in cost-effectiveness outcomes between China and the United States arise not only from drug pricing disparities but also from fundamental differences in their healthcare systems.

In health-related cost-effectiveness analyses, the WTP threshold represents the estimated amount that consumers are willing to pay for health benefits and is often used as a non-monetary measure of the cost-effectiveness of health interventions(48, 49), inherently reflecting each society's capacity and preference for financing health-care resource allocation. Given systematic differences in national conditions and social preferences, these thresholds are not strictly fair or comparable. In economic decision-making, when the ICER is below the relevant WTP threshold, the intervention is generally considered cost-effective. In this study, we used the conventional US threshold of $150,000/QALY, as commonly applied by the US Institute for Clinical and Economic Review, although the World Health Organization has suggested that values of up to 3 times the GDP per capita may be justified(49, 50). It is worth mentioning that the use of a higher WTP threshold ($257,430/QALY, three times the US per capita GDP in 2024) did not result in osimertinib becoming the most cost-effective strategy in the USA, although it did improve the cost-effectiveness. In China, due to the uneven and insufficient economic development of different parts of the country, there are significant differences in the per capita GDP among different economic regions. In 2024, the WTP thresholds in Beijing (eastern region), Hubei (central), Liaoning (northeastern), and Gansu (western) were $94,443, $42,564, $32,383, and $21,865/QALY, respectively (51). At a WTP threshold of $39,632/QALY, osimertinib thus remains the most cost-effective treatment option in most provinces in eastern and central China, while placebo may be a more suitable choice in the less developed northeastern and western regions. Nevertheless, this does not mean that these patients should be treated with less effective therapeutic options. The healthcare systems and drug pricing policymakers should establish assistance strategies and insurance policies based on differences in the economic development of different regions.

This study has several significant strengths. Firstly, it used the latest clinical evidence to evaluate the cost-effectiveness of osimertinib vs. placebo in patients with stage III unresectable EGFR-mutated NSCLC following chemoradiotherapy. Secondly, the use of treatment strategies targeting disease-specific molecular targets has become indispensable in lung cancer, greatly advancing precision medicine and personalized treatment (52). This study analyzed the economic outcomes of different patient subgroups, providing strong guidance for personalized management and treatment decisions based on the characteristics of specific patient subgroups. In addition, considering the differences between healthcare systems in different countries, to enhance the applicability of the results, the study evaluated cost-effectiveness from the perspectives of a high-income country (the USA) and a middle-income country (China), providing scientific references to guide health-related decisions in countries with varying income levels.

The study also has several limitations. Due to the relatively short follow-up time of the Phase 3 LAURA clinical trial, survival data may change with the prolongation of the follow-up, particularly in the median OS data, and the extrapolated survival estimates are likely to be uncertain. Thus, despite conducting sensitivity and subgroup analyses to assess this uncertainty, it is an inevitable limitation of our model that requires updated data from the LAURA trial for validation. Second, since the LAURA trial did not include information on HRQOL, health utility data for PFS and PD were obtained from the literature, which may have introduced bias into the results of the model, although the results of subsequent scenario analysis based on health utilities showed osimertinib vs. placebo remained the relatively most cost-effective option in the eastern economic regions of China. Third, consistent with most cost-effectiveness analyses, the use of the same virtual patient cohort with different national healthcare systems due to the absence of data is an inescapable issue in clinical trial-based cost-effectiveness analyses, even if the trial showed similar trends in terms of safety and efficacy in subgroups and the population as a whole. Fourth, placebo was selected as the control group, which may not be representative of the real-world practice for typical international patients with unresectable stage III EGFR-mutated NSCLC. However, it is particularly relevant for populations who are PD-L1 negative, have contraindications to immunotherapy, or are unable to afford costly treatments—especially given the current limited evidence indicating that the benefits of consolidation therapy with immunotherapy for patients with unresectable stage III EGFR-mutated NSCLC remain uncertain (16). Finally, without the availability of separate survival curves based on patient subgroups, economic estimates may show differences from real-life survival outcomes by using specific HRs, requiring additional follow-up studies on these subgroups.

## 5 Conclusion

In China, treatment with osimertinib rather than placebo appears to be an effective and economically accessible option for patients with stage III EGFR-mutated NSCLC with no disease progression during or after chemoradiotherapy, especially in the eastern and central economic regions of China. However, this does not apply to the USA currently.

## Data Availability

The original contributions presented in the study are included in the article/Supplementary material, further inquiries can be directed to the corresponding authors.

## Supplementary material

Supplementary Figure S1 Model Structure.

Supplementary Figure S2 AIC and BIC statistics for alternate parametric survival distributions.

Supplementary Figure S3 Probability Sensitivity Analysis Scatter Plot.

Supplementary Table S1 The CHEERS 2022 checklist.

Supplementary Table S2 Details of Treatment Strategy and Unit Costs.

Supplementary Table S3 Summary of Statistical Goodness-of-fit of K-M Curve.

Supplementary Table S4 Scenario analysis for health utilities.
