# Supplementary material for: International cost-effectiveness analysis in osimertinib after chemoradiotherapy in stage III EGFR-mutated non-small cell lung cancer
Source: Front Public Health. 2025 Oct 29;13:1698562. doi: 10.3389/fpubh.2025.1698562 (PMC12605223; doi:10.3389/fpubh.2025.1698562)
Supplement: Supplementary Figure S1 — Model Structure. [file Supplementary_file_1.docx]

***Supplementary Materials***

**Figure S1.** Model Structure.

**Figure S2.** AIC and BIC statistics for alternate parametric survival distributions.

**Figure S3.** Probability Sensitivity Analysis Scatter Plot.

**Table S1.** The CHEERS 2022 checklist.

**Table S2.** Details of Treatment Strategy and Unit Costs.

**Table S3.** Summary of Statistical Goodness-of-fit of K-M Curve.

**Table S4.** Scenario analysis for health utilities.

**Figure S1. Model Structure.**


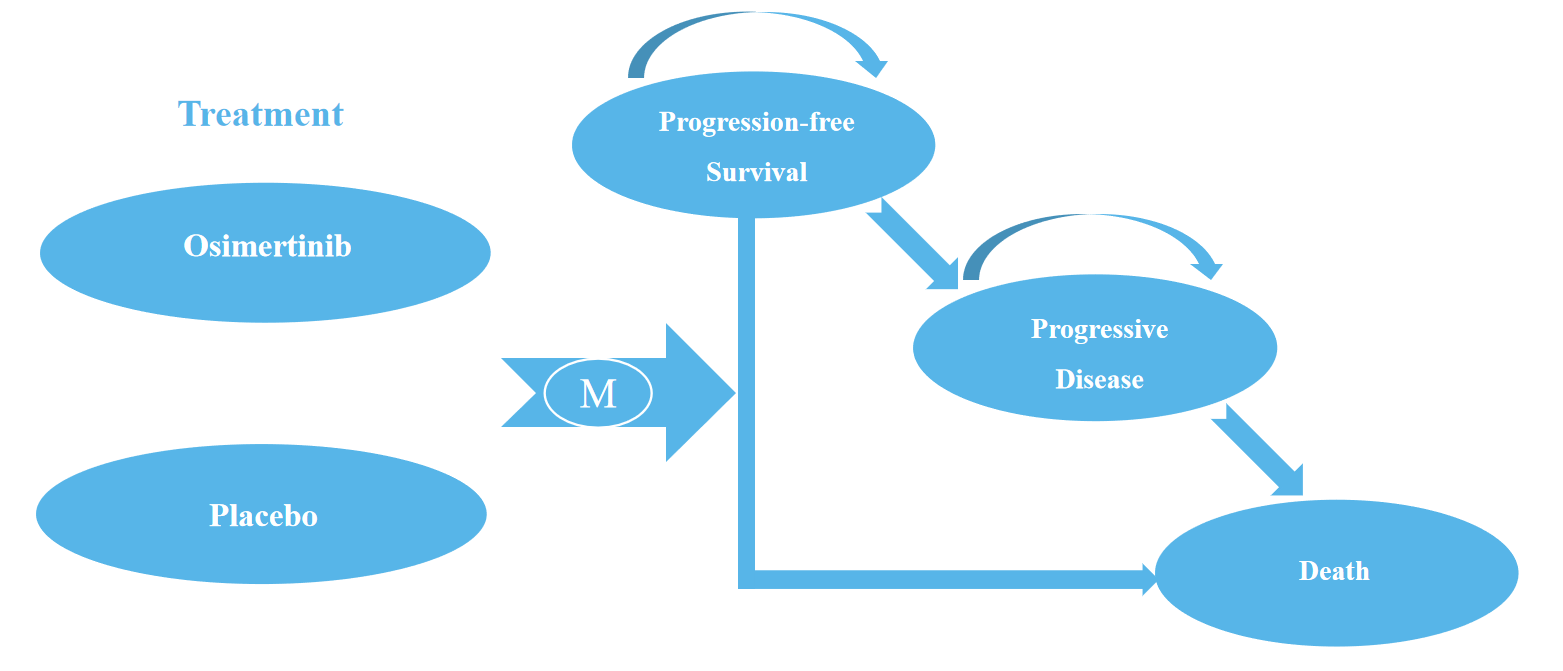


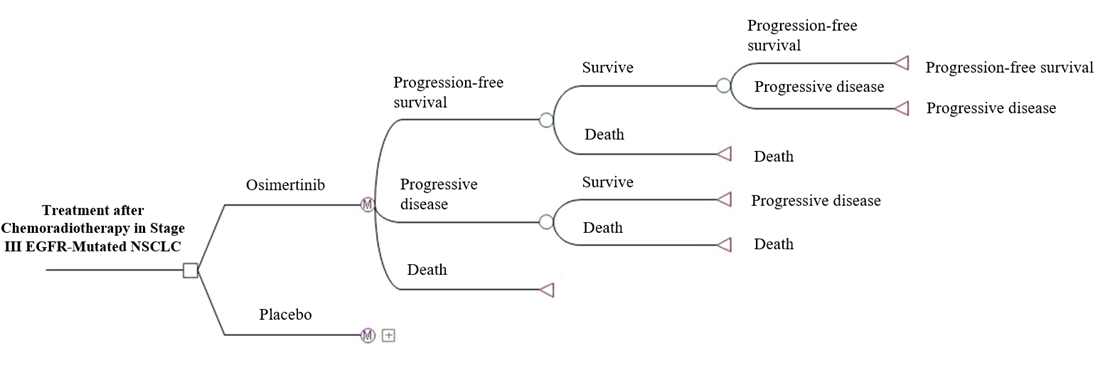


Abbreviation: NSCLS, non-small cell lung cancer; M, Markov.

**Figure S2. AIC and BIC statistics for alternate parametric survival distributions.**


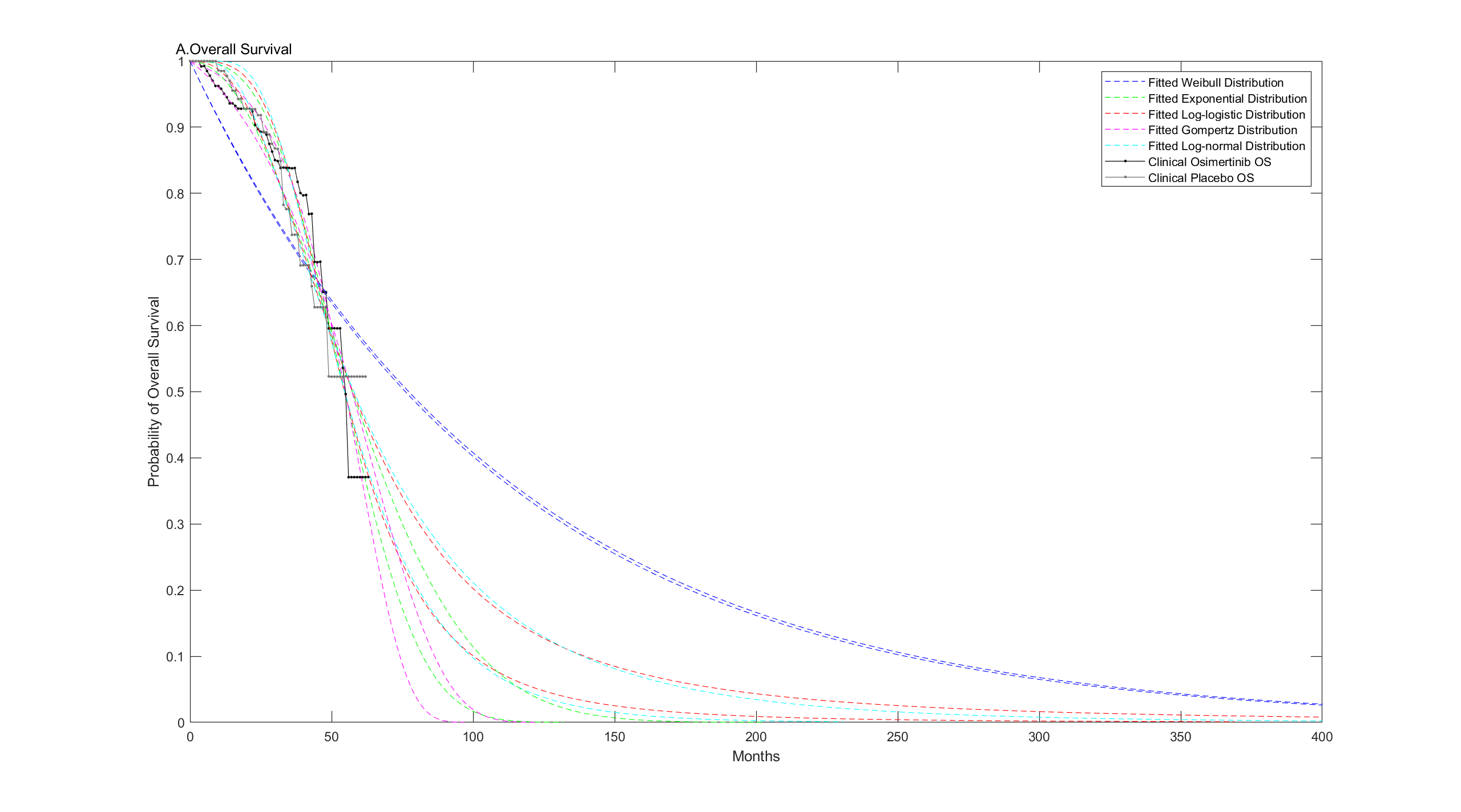


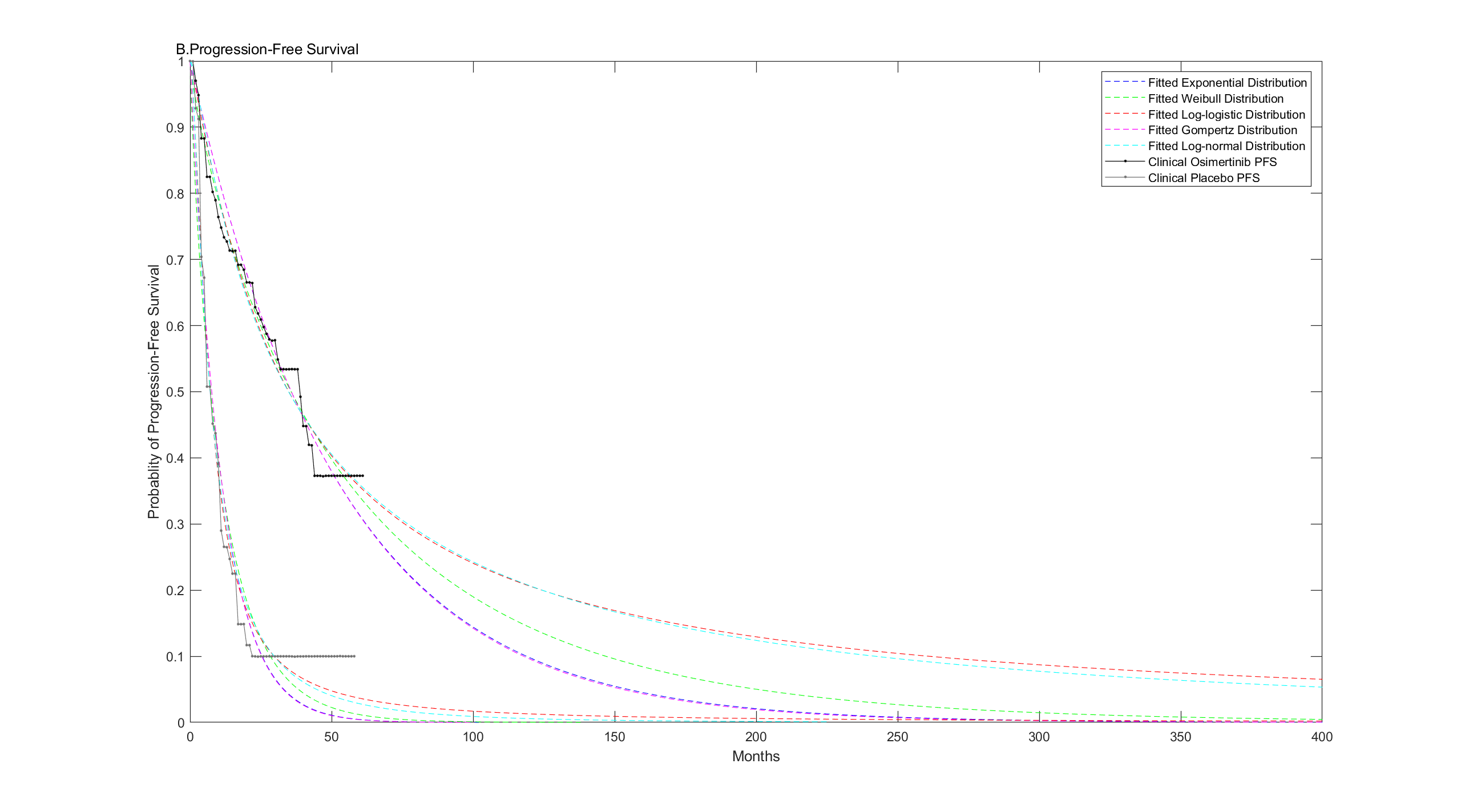


Abbreviation: OS, overall survival; PFS, progression-free survival. Kaplan-meier curve fitting and extrapolation for overall survival (A) and progression-free survival (B), respectively.

**Figure S3.** **Probability Sensitivity Analysis Scatter Plot.**


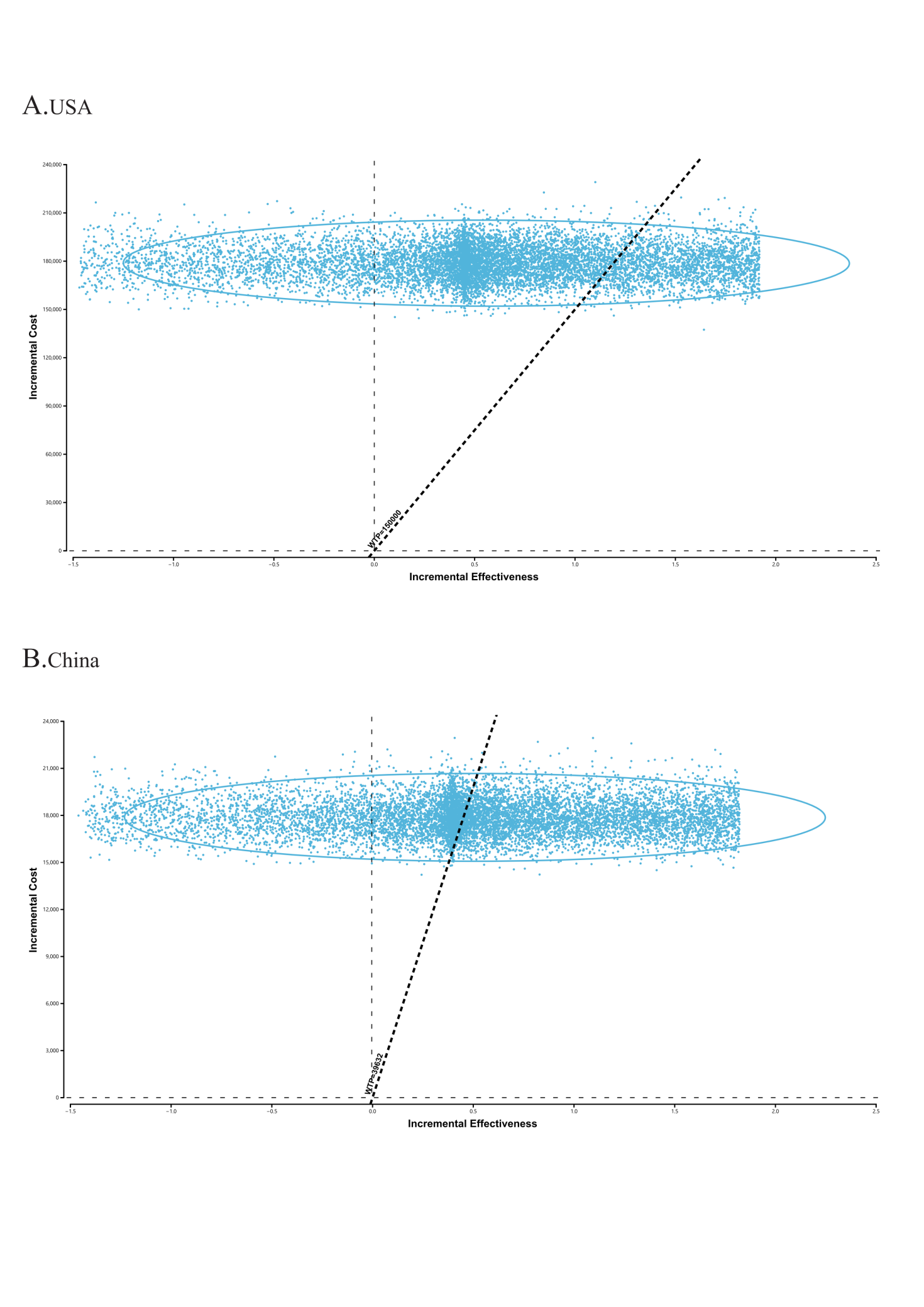


Abbreviation: WTP, willingness-to-pay.

Each blue dot in the diagram represents a simulation result of 10,000 Monte Carlo simulations. The ellipse represents the 95% confidence intervals, and the dashed lines represent the willingness-to-pay thresholds. Below thedashed line represents the probability that osimertinib will be cost-effective.

**Table S1. The CHEERS 2022 checklist.**

| **Section/item** | **Item No** | **Recommendation** | **Reported?** |
| --- | --- | --- | --- |
| **Title and abstract** | | | |
| Title | 1 | Identify the study as an economic evaluation and specify the interventions being compared. | Yes |
| Abstract | 2 | Provide a structured summary that highlights context, key methods, results, and alternative analyses. | Yes |
| **Introduction** | | | |
| Background and objectives | 3 | Give the context for the study, the study question, and its practical relevance for decision making in policy or practice. | Yes |
| **Methods** | | | |
| Health economic analysis plan | 4 | Indicate whether a health economic analysis plan was developed and where available. | Yes |
| Study population | 5 | Describe characteristics of the study population (such as age range, demographics, socioeconomic, or clinical characteristics). | Yes |
| Setting and location | 6 | Provide relevant contextual information that may influence findings. | Yes |
| Comparators | 7 | Describe the interventions or strategies being compared and why chosen. | Yes |
| Perspective | 8 | State the perspective(s) adopted by the study and why chosen. | Yes |
| Time horizon | 9 | State the time horizon for the study and why appropriate. | Yes |
| Discount rate | 10 | Report the discount rate(s) and reason chosen. | Yes |
| Selection of outcomes | 11 | Describe what outcomes were used as the measure(s) of benefit(s) and harm(s) | Yes |
| Measurement of outcomes | 12 | Describe how outcomes used to capture benefit(s) and harm(s) were measured. | Yes |
| Valuation of outcomes | 13 | Describe the population and methods used to measure and value outcomes. | Yes |
| Measurement and valuation of resources and costs | 14 | Describe how costs were valued. | Yes |
| Currency, price date, and conversion | 15 | Report the dates of the estimated resource quantities and unit costs, plus the currency and year of conversion. | Yes |
| Rationale and description of model | 16 | If modelling is used, describe in detail and why used. Report if the model is publicly available and where it can be accessed. | Yes |
| Analytics and assumptions | 17 | Describe any methods for analysing or statistically transforming data, any extrapolation methods, and approaches for validating any model used. | Yes |
| Characterizing heterogeneity | 18 | Describe any methods used for estimating how the results of the study vary for subgroups. | Yes |
| Characterizing distributional effects | 19 | Describe how impacts are distributed across different individuals or adjustments made to reflect priority populations. | Yes |
| Characterizing uncertainty | 20 | Describe methods to characterise any sources of uncertainty in the analysis. | Yes |
| Approach to engagement with patients and others affected by the study | 21 | Describe any approaches to engage patients or service recipients, the general public, communities, or stakeholders (such as clinicians or payers) in the design of the study. | Not applicable |
| **Results** | | | |
| Study parameters | 22 | Report all analytic inputs (such as values, ranges, references) including uncertainty or distributional assumptions. | Yes |
| Summary of main results | 23 | Report the mean values for the main categories of costs and outcomes of interest and summarise them in the most appropriate overall measure. | Yes |
| Effect of uncertainty | 24 | Describe how uncertainty about analytic judgments, inputs, or projections affect findings. Report the effect of choice of discount rate and time horizon, if applicable. | Yes |
| Effect of engagement with patients and others affected by the study | 25 | Report on any difference patient/service recipient, general public, community, or stakeholder involvement made to the approach or findings of the study. | Not  applicable |
| **Discussion** | | | |
| Study findings, limitations, generalizability, and current knowledge | 26 | Report key findings, limitations, ethical or equity considerations not captured, and how these could affect patients, policy, or practice. | Yes |
| **Other** | | | |
| Source of funding | 27 | Describe how the study was funded and any role of the funder in the identification, design, conduct, and reporting of the analysis | Yes |
| Conflicts of interest | 28 | Report authors conflicts of interest according to journal or International Committee of Medical Journal Editors requirements. | Yes |

Reference:

*Husereau D, Drummond M, Augustovski F, de Bekker-Grob E, Briggs AH, Carswell C, et al. Consolidated Health Economic Evaluation Reporting Standards 2022 (CHEERS 2022) Statement: Updated Reporting Guidance for Health Economic Evaluations. Value Health. 2022;25(1):3-9.*

**Table S2. Details of Treatment Strategy and Unit Costs.**

| **Drug** | **Dose** | **Time** | **Unit price ($)** | **Cost per cycle ($)** |
| --- | --- | --- | --- | --- |
| Osimertinib (The United States) | Osimertinib 80 mg | Administered osimertinib 80 mg once daily | 7.5142 | 18,034 |
| Osimertinib (China) | Osimertinib 80 mg | Administered osimertinib 80 mg once daily | 0.2855 | 685 |
| Placebo | Placebo 80 mg | Administered placebo 80 mg once daily | 0 | 0 |

**Table S3. Summary of Statistical Goodness-of-fit of K-M Curve.**

|  | **Exponential** | **Weibull** | **Gompertz** | **Log-logistic** | **Log-normal** |
| --- | --- | --- | --- | --- | --- |
| **Osimertinib OS curve** | | | | | |
| AIC | 38.8502 | 38.7258 | 40.0259 | 38.1573 | 37.8265 |
| BIC | 43.168 | 40.8847 | 44.3436 | 42.4751 | 42.1442 |
| **Placebo OS curve** | | | | |  |
| AIC | 38.0368 | 37.9171 | 38.4658 | 37.7366 | 37.4287 |
| BIC | 40.180 | 42.2033 | 42.7521 | 42.0229 | 41.7149 |
| **Osimertinib PFS curve** | | | | | |
| AIC | 77.1207 | 76.942 | 77.2491 | 76.6944 | 76.5901 |
| BIC | 81.3749 | 81.0092 | 81.5034 | 80.9487 | 80.8444 |
| **Placebo PFS curve** | | | | | |
| AIC | 311.4115 | 274.8626 | 316.2673 | 244.4964 | 250.0413 |
| BIC | 313.4891 | 279.0177 | 320.4223 | 248.6515 | 254.1964 |

Abbreviation: OS, overall survival; PFS, progression-free survival; AIC, Akaike’s information criterion; BIC, Bayesian information criterion.

According to the AIC and BIC value of the distributions and the visual fitting of the curves (Figure S2) showed that the Weibull distributionisprobably the most reasonable parametric survival model. Weibull distribution is flexible and widely used for matching patients with three statesover time, because it can monotonously increase or decrease risk functions, and it is suitable for estimating events occurring during early follow-up work.

**Table S4.** Scenario analysis for health utilities.

| **Scenario** | **PFS Utility** | **PD Utility** | **ICER $/QALY** | | **Comments** | |
| --- | --- | --- | --- | --- | --- | --- |
|  |  |  | **The United States** | **China** | **The United States** | **China** |
| Baseline | 0.791 | 0.653 | 322,308 | 35,186 | Not cost-effective | Cost-effective |
| Scenario A | 0.949 | 0.784 | 268,747 | 29,340 | Not cost-effective | Cost-effective |
| Scenario B | 0.949 | 0.522 | 170,237 | 181,89 | Not cost-effective | Cost-effective |
| Scenario C | 0.633 | 0.522 | 402,533 | 43,942 | Not cost-effective | Not cost-effective |

Abbreviation: PFS, progression-free survival; PD, progressive disease.
